# The effectiveness and safety of curcumin as a complementary therapy in inflammatory bowel disease: A protocol of systematic review and meta-analysis

**DOI:** 10.1097/MD.0000000000022916

**Published:** 2020-10-23

**Authors:** Zhenhuan Yang, Wenjing Liu, Xuefeng Zhou, Xiaoran Zhu, Feiya Suo, Shukun Yao

**Affiliations:** a Graduate School, Beijing University of Chinese Medicine; b Department of Gastroenterology; c Department of Dermatology and Venerology; d Beijing Key Laboratory for Immune-Mediated Inflammatory Disease, Institute of Clinical Medical Sciences, China-Japan Friendship Hospital, Beijing, China.

**Keywords:** curcumin, inflammatory bowel diseases, protocol, systematic review

## Abstract

**Background:**

Inflammatory bowel diseases (IBD), which include Crohn disease and ulcerative colitis, affect several million individuals worldwide. Curcumin as a complementary therapy has been used to cure the IBD, yet the efficacy and safety of curcumin remains to be assessed. In this study, we aim to draw up a protocol for systematic review to evaluate the efficacy and safety of curcumin for IBD.

**Methods:**

We will search the following electronic databases from inception to September 31, 2020: PubMed, Cochrane Library, EMBASE, Web of Science, Medline, the China National Knowledge Infrastructure Database, Wan Fang Database, the Chinese Scientific Journal Database, and Chinese Biomedical Literature Database. Clinical trial registrations, potential gray literatures, relevant conference abstracts and reference list of identified studies will also be searched. Relevant randomized controlled clinical trials were enrolled and analyzed. The literature selection, data extraction, and quality assessment will be completed by 2 independent authors. Either the fixed-effects or random-effects model will be used for data synthesis based on the heterogeneity test. Clinical remission will be evaluated as the primary outcome. Clinical response, endoscopic remission, inflammatory markers and adverse events will be assessed as the secondary outcomes. The RevManV.5.3.5 will be used for Meta-analysis. Subgroup analyses of doses, delivery way, frequency of treatment and the degree of IBD severity or different forms of IBD were also conducted.

**Results:**

This study will provide a synthesis of current evidence of curcumin for IBD from several aspects, such as clinical remission, clinical response, endoscopic remission, inflammatory markers, and adverse events.

**Conclusion:**

The conclusion of our study will provide updated evidence to judge whether curcumin is an effective solution to IBD patients.

**INPLASY registration number:**

INPLASY202090065.

## 1 Introduction

Inflammatory bowel diseases (IBD), which include Crohn disease (CD)and ulcerative colitis (UC), affect more than 3.5 million people, and their incidence is increasing worldwide.^[1]^ These diseases are characterized by debilitating and chronic relapsing and remitting inflammation of the gastrointestinal tract (for CD) or the colon (in UC).^[2]^ The pathogenesis is multifactorial, involving genetic predisposition, epithelial barrier defects, dysregulated immune responses, and environmental factors.^[3]^ IBD are attracting increased attention at present for a number of reasons.

The conventional approach to IBD aims to induce and maintain clinical remission free of corticosteroids, thus minimizing the impact on quality of life.^[4]^ Currently, treatment options for both diseases, including drugs such as 5-aminosalicylates,^[5]^ corticosteroids,^[6]^ anti-tumor necrosis factor alpha drugs,^[7]^ antibiotics,^[8]^ probiotics,^[9]^ and immunosuppressants,^[10]^ are costly, often involve significant side effects, and are limited in effectiveness and specificity. Studies indicate that a substantial proportion of patients fail to respond to mesalamine for remission induction will often relay to corticosteroids and/or immunomodulators to control the disease.^[11]^ Corticosteroid resistance/refractoriness rates range from 8.9% to 25% in individuals with IBD.^[12]^ Despite an early and wide use of immunosuppressants and biologics, approximately one half of the patients with Crohn disease still needed bowel resection within 10 years after the diagnosis.^[13]^ Patients with long-standing inflammatory bowel disease (IBD) involving at least 1/3 of the colon are at increased risk for colorectal cancer (CRC).^[14]^ Therefore, the need of new compounds that can induce and maintain remission in IBD without serious side effects is around the corner.

Curcumin, a polyphenol derived from the turmeric plant (Curcuma longa),^[15]^ has been demonstrated to hold many health-related benefits and pharmacological effects, including anti-inflammatory, anti-oxidant, immunomodulatory effects, as well as its potential to alter the intestinal microbiome.^[16–18]^ Curcumin as a complementary therapy in ulcerative colitis has become quite common, two randomized, controlled trials investigated the efficacy and safety of curcumin in patients with crohn's disease recently.^[19–27]^ The mechanism of its anti-inflammatory action deemed to be the most relevant is the inhibition of NF-κB, by blocking IκB kinase, thereby inhibiting the expression of pro-inflammatory cytokines (IL-1, IL-6, and TNF-α.^[28]^ Therefore, it is necessary to conduct a meta-analysis of curcumin for IBD. In this study, we aim to analyze the studies published so far, to review the positive or negative effects of the use of curcumin, and to determine whether it is safe and effective as a complementary therapy in the management of IBD.

## 2 Methods

### 2.1 Study registration

The systematic review protocol has been registered on the International Platform of Registered Systematic Review and Meta-Analysis Protocols (INPLASY). The registration number was INPLASY202090065 (DOI number is 10.37766/inplasy2020.9.0065, https://inplasy.com/inplasy-2020-9-0065/). This meta-analysis is a secondary research which based on some previously published data. Therefore, the ethical approval or informed consent was not required in this study.

### 2.2 Inclusion criteria for study selection

#### 2.2.1 Types of studies

All relevant randomized controlled trials (RCTs) in English and Chinese will be included. Duplicated publications, review articles, animal studies, editorial letters, in-vitro studies, observational, and descriptive studies, such as case reports and case series will be excluded.

#### 2.2.2 Types of participants

Study participants in different age ranges with IBD can be included in the study without restricting nationality, sex, race, occupation, or education.

#### 2.2.3 Types of interventions

The control group: basic treatment (including conventional medication and health education) and Placebo. The experimental group: basic treatment and curcumin.

#### 2.2.4 Types of outcome measures

##### 2.2.4.1 Primary outcomes

Clinical remission will be evaluated as the primary outcome.

##### 2.2.4.2 Secondary outcomes

The secondary outcomes of this review will include:

1. Clinical response
2. Endoscopic remission
3. Inflammatory markers (hs-CRP, ESR, inflammatory factor levels)
4. Adverse events.

### 2.3 Search methods for identification of studies

#### 2.3.1 Electronic searches

We will search the following electronic databases from inception to September 31, 2020: PubMed, Cochrane Library, EMBASE, Web of Science, Medline, the China National Knowledge Infrastructure Database, Wan Fang Database, the Chinese Scientific Journal Database, and Chinese Biomedical Literature Database. The search strategy for Medline (via PubMed) is shown in Table 1, other electronic databases will also be searched based on this strategy.

**Table 1:**
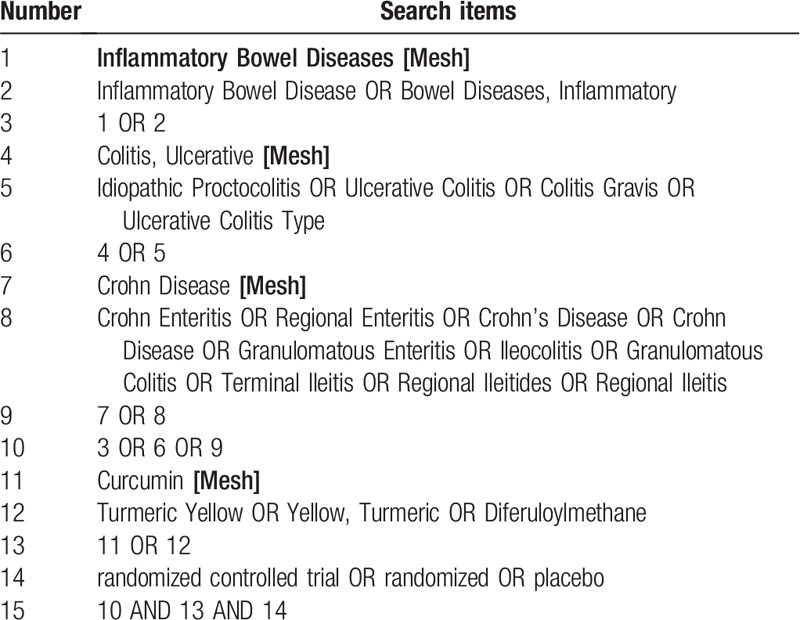
Search strategy for Medline (via PubMed).

#### 2.3.2 Searching other resources

The reference lists of studies and systematic reviews will be examined and retrieved for additional trials. Potential gray literatures will be searched in OpenGrey.eu. We will search relevant conference abstracts for eligible trials. In addition, we will also search the WHO International Clinical Trials Registry Platform and the ClinicalTrials.gov for all new reviews relevant to this topic.

### 2.4 Data collection and analysis

#### 2.4.1 Selection of studies

**Figure 1:**
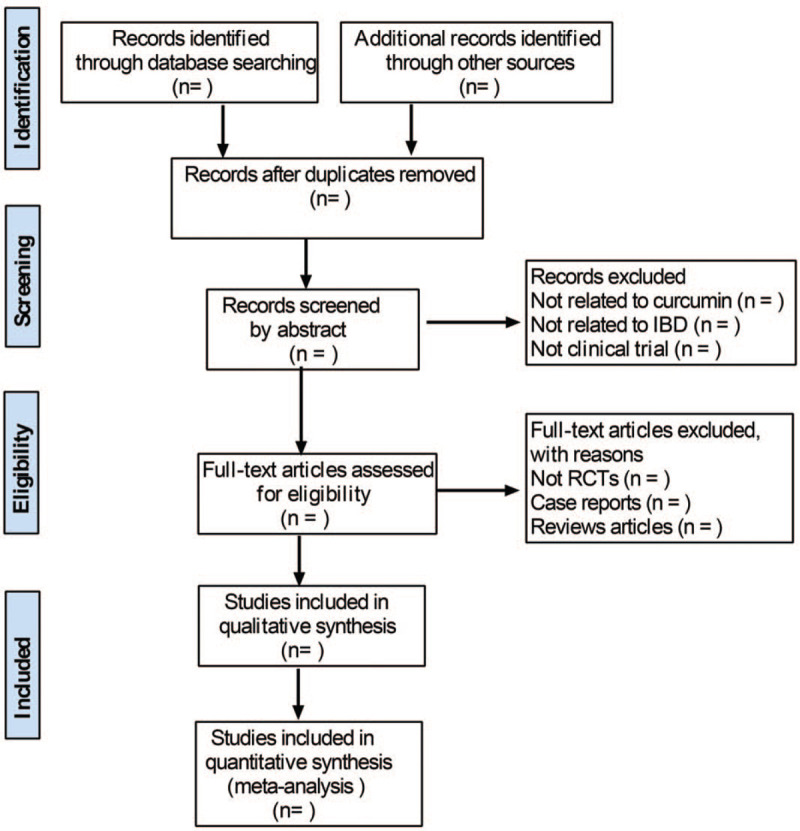
Study selection process for the meta-analysis.

We search the specified databases to obtain relevant literature, import them into a database created by Endnote X8 and screen out the duplicate documents. Two review authors will independently screen the titles, abstracts, and keywords of all retrieved records. If necessary, full texts will be examined according to the inclusion criteria for further assessment. We will resolve disagreements by discussing with the third author. The screening flow diagrams of this study will be shown in Figure 1.

#### 2.4.2 Data extraction and management

We will make a standard data collection sheet before data extraction. Two reviewers will independently extract data from the selected studies and fill in the data collection sheet. Discrepancies and uncertainties will be resolved by consensus between the 2 review authors or by asking the third author to make a final decision. We will extract the following data:

1. General information: the first author, title, the journal, publication type, publication year, country.
2. Methods: study design, sample size, randomization, allocation concealment, blinding methods, inclusion criteria, and exclusion criteria.
3. Participants: age, gender, severity of IBD, baseline disease activity.
4. Interventions: type of control, duration of treatment, frequency of treatment.
5. Outcomes: primary and secondary outcomes, adverse effects, and follow up.

#### 2.4.3 Assessment of bias in the included studies

Two review authors will independently use the criteria outlined in the Cochrane Handbook for Systematic Reviews of Interventions to assess the risk of bias in the included studies. The following 6 domains in the Cochrane “Risk of bias tool” will be assessed: random sequence generation, allocation concealment, blinding, incomplete outcome data, selective reporting, and other bias. We will grade each potential trial of bias as high, low, and unclear. Any disagreement will be resolved by discussing or by asking the 3rd author to make a final decision.

#### 2.4.4 Measures of treatment effect

The continuous data will be expressed as mean difference (MD) or standard MD (SMD) with 95% confidence intervals (CIs), and the dichotomous outcomes will be estimated by the risk ratio (RR) with 95% CIs.

#### 2.4.5 Unit of analysis issues

In order to avoid carryover effects, we will only extract the first experimental period data of cross-over trials. For trials with multiple intervention groups, we will combine all relevant control intervention groups and experimental intervention groups of the trial into a single group to avoid a unit-of-analysis error.

#### 2.4.6 Management with missing data

For missing data, we will try to contact the original author to obtain the relevant data by email or phone. If we are unable to obtain missing data, the analysis will base on available data.

#### 2.4.7 Assessment of heterogeneity

Heterogeneity will be assessed by visual inspection of the forest plots and detected by standard Chi-squared test and I^2^ statistic. I^2^ < 50% will be considered that the included studies are homogeneous, while I^2^ > 50% will be taken as evidence of representing substantial heterogeneity. Sensitivity analysis and subgroup analysis will be selected to detect the possible reason of substantial heterogeneity.

#### 2.4.8 Assessment of reporting bias

When the number of included studies is more than 10, the funnel plot will be used to detect the reporting bias.

#### 2.4.9 Data synthesis

RevManV.5.3.5 will be used for data analysis and synthesis. Continuous data will be expressed as MD/SMD with 95% CIs, while the dichotomous outcomes will be presented as RR with 95% CIs. When I^2^ < 50%, the fixed effect model will be adopted to analyze. Otherwise, the random effect model will be selected. Additionally, we will use the sensitivity analysis and subgroup analysis to explore the causes of heterogeneity.

#### 2.4.10 Subgroup analysis

Subgroup analysis will be carried out based on the age of patients, different types of curcumin therapies, duration of treatment, frequency of treatment and the degree of IBD severity or different types of IBD.

#### 2.4.11 Sensitivity analysis

If there are sufficient studies included, we will take sensitivity analyses to test the robustness and reliability of the results. The sensitivity analysis focuses on research characteristics or types such as methodological quality, and examines the effects of total effects by excluding certain low quality studies or unblinded studies.

## 3 Discussion

Curcumin, with the advantages of low-cost, reliable sources and low side effects, is holding considerable attention for IBD treatment. Curcumin, also known as diferuloylmethane, has been a popular supplement largely because of its affordability and safety, with no known toxic side effects in humans up to doses of 12 g/day.^[29]^ Its significant NF-κB inhibition, expressed by regulating NF-κB/IκB pathway and down-regulating expression of pro-inflammatory cytokines, was thought to be the key mechanism for the anti-inflammatory.^[30–32]^ In AP-1 signaling, curcumin can inhibit MAPK, ERK1/2, JNK, and p38, both directly and indirectly, thereby limiting transcription of inflammatory target genes.^[33]^ We hope this systematic review will provide more reliable evidence in the management of IBD.

There are still some limitations in this review. Limited by our language capability, we just collect studies in English and Chinese. Meanwhile, the dose, delivery way, intervention time, and form of curcumin were different among them, which might add bias to our study outcomes.

## Author contributions

**Conceptualization:** Zhenhuan Yang, Wenjing Liu, Shukun Yao

**Data curation:** Xiaoran Zhu, Feiya Suo

**Formal analysis:** Wenjing Liu

**Project administration:** Zhenhuan Yang, Shukun Yao

**Supervision:** Xuefeng Zhou, Xiaoran Zhu, Feiya Suo

**Writing – original draft:** Zhenhuan Yang, Wenjing Liu

**Writing – review & editing:** Zhenhuan Yang, Shukun Yao
